# Context-Dependent Requirement for dE2F during Oncogenic Proliferation

**DOI:** 10.1371/journal.pgen.1000205

**Published:** 2008-10-03

**Authors:** Brandon N. Nicolay, Maxim V. Frolov

**Affiliations:** Department of Biochemistry and Molecular Genetics, University of Illinois at Chicago, Chicago, Illinois, United States of America; Harvard Medical School, Howard Hughes Medical Institute, United States of America

## Abstract

The Hippo pathway negatively regulates the cell number in epithelial tissue. Upon its inactivation, an excess of cells is produced. These additional cells are generated from an increased rate of cell division, followed by inappropriate proliferation of cells that have failed to exit the cell cycle. We analyzed the consequence of inactivation of the entire E2F family of transcription factors in these two settings. In *Drosophila*, there is a single activator, dE2F1, and a single repressor, dE2F2, which act antagonistically to each other during development. While the loss of the activator dE2F1 results in a severe impairment in cell proliferation, this defect is rescued by the simultaneous loss of the repressor dE2F2, as cell proliferation occurs relatively normally in the absence of both dE2F proteins. We found that the combined inactivation of dE2F1 and dE2F2 had no significant effect on the increased rate of cell division of Hippo pathway mutant cells. In striking contrast, inappropriate proliferation of cells that failed to exit the cell cycle was efficiently blocked. Furthermore, our data suggest that such inappropriate proliferation was primarily dependent on the activator, *de2f1*, as loss of *de2f2* was inconsequential. Consistently, Hippo pathway mutant cells had elevated E2F activity and induced dE2F1 expression at a point when wild-type cells normally exit the cell cycle. Thus, we uncovered a critical requirement for the dE2F family during inappropriate proliferation of Hippo pathway mutant cells.

## Author Summary

The E2F transcription factor family is considered to be the best-characterized downstream target of the retinoblastoma protein (pRB). The pRB pathway is functionally inactivated in most tumor cells, and it is thought that unrestrained activity of E2F drives inappropriate proliferation in tumors. We utilized the relative simplicity of the *Drosophila* model to determine the role of the dE2F family in proliferation of cells following inactivation of the recently identified Hippo tumor suppressor pathway. We found that Hippo pathway mutant cells require the dE2F family to delay the cell cycle exit and to proliferate inappropriately when wild-type cells enter quiescence. This is significant since the loss of the entire dE2F family exerts almost no effect on the ability of Hippo pathway mutations to accelerate proliferation of actively dividing cells. Thus, the importance of the dE2F family in cells with an inactivated tumor suppressor pathway varies in different contexts. This discovery may have implications in designing anti-cancer therapies that inhibit E2F activity.

## Introduction

The Retinoblastoma tumor suppressor protein (pRB) and the related proteins p107 and p130 negatively regulate cell proliferation. In a textbook model, the role of pRB family members in cell cycle regulation is explained by their ability to attenuate the activity of E2F transcription factors. E2F is best known for its ability to control the G1/S transition and is rate limiting for S phase entry (for review see: [1]–[4]). The E2F transcriptional program provides cell cycle dependent expression of a large panel of genes encoding replication proteins, cell cycle regulators and others. In early G1 phase, members of the pRB family are complexed with members of the E2F family and repress expression of E2F regulated genes through recruitment of corepressor complexes to target promoters. In late G1 phase, cyclin dependent kinases phosphorylate pRB family members, thus releasing free E2F proteins to allow induction of E2F-dependent transcription. Since functional inactivation of the pRB pathway occurs in most tumor cells it is thought that unrestrained E2F activity drives inappropriate proliferation in tumors [5]. Such an idea is further supported by findings that mutations in E2f genes reduce proliferation in *Rb* deficient mouse embryos [6]–[8].

In mammalian cells, E2F activity is a combined output of eight family members, which, in turn, are loosely grouped into a class of repressors (E2F-3b through E2F-8) and a class of activators (E2F-1 through E2F3a). E2F-1 through E2F-6 require a heterodimeric partner of the DP family of proteins to bind to DNA, while E2F-7 and E2F-8 bind to DNA in a DP-independent manner. As a way to dissect the contribution of E2F to cell proliferation, dominant negative forms of DP and E2F, dn-DP and dn-E2F respectively, were used. Expression of dn-E2F, which binds to DNA, but fails to repress or activate, leads to immortalization in mouse fibroblasts and renders cells resistant to senescence induced by p19^ARF^, p53 or by RAS^V12^ [9]. However, cells expressing dn-E2F were impaired in the ability to proliferate following serum stimulation. This suggests that E2F activity is not needed during cell proliferation but is required in a specific context, such as cell cycle re-entry from quiescence. In contrast, a reduction of DP function, either by siRNA or by using a dn-DP form, resulted in cell cycle arrest and a senescence-like phenotype, indicating that E2F is in fact needed for cell proliferation [10],[11]. One potential explanation for these discrepancies is that reducing DP does not inactivate the total pool of E2Fs, since E2F-7 and E2F-8 repressors bind to DNA in a DP-independent manner, and therefore the two remaining E2Fs may induce the cell cycle arrest. An alternative explanation is that dn-E2F does not completely inhibit E2F activity [11] and the remaining E2F activity is sufficient to sustain cell proliferation.

The biological role of E2F in the context of animal development is being extensively studied by using gene targeting approaches in mice. However, interpretation of the phenotypes of individual E2F knockouts is often complicated by the redundancy and compensation among the family members. The impact of genetic ablation of E2f genes on cell proliferation is more profound in compound knockouts. Mouse embryonic fibroblasts (MEFs) lacking a whole class of activator E2Fs, *E2f-1, E2f-2* and *E2f-3*, fail to proliferate due to a high level of p21 [12], *E2f-4*; *E2f-5* double knockouts are defective in a p16 mediated cell cycle arrest [13], while *E2f-7*; *E2f-8* knockouts have a high level of apoptosis due to deregulation of *E2f-1* expression [14]. Nevertheless, the large number of E2F genes makes it currently unfeasible to genetically ablate all E2F activity in mammals to determine the consequences of the loss of E2F function on cell proliferation.

Genetically, *Drosophila* provides a relatively simpler system to study the role of E2F, since the corresponding families are smaller. The *Drosophila* E2F family consists of a single activator, dE2F1, and a lone repressor, dE2F2. Both dE2Fs dimerize with the single dDP protein in order to bind to DNA. Unlike mammalian cells, the *Drosophila* genome lacks orthologs of E2F-7 or E2F-8 that bind to DNA in a dDP independent manner. It is important to note that the loss of *dDP* has been shown to functionally inactivate both dE2F1 and dE2F2 [15]. A *de2f1* mutation severely reduces cell proliferation, leads to the loss of expression of E2F target genes, and almost complete cessation of DNA synthesis [16],[17]. Strikingly, these defects are largely suppressed by a concomitant mutation in *de2f2*. *de2f1 de2f2* double mutant animals can survive until late pupal stages and show normal patterns of cell proliferation and differentiation even though E2F targets are no longer expressed in a cell cycle dependent manner and are likely to be present at suboptimal levels [18]. A similar phenotype has been observed in *dDP* mutant animals. Thus, the complete loss of E2F function in *dDP* or in *de2f1 de2f2* mutants is permissive for cell proliferation and appears to have a relatively minor impact on animal development; however, whether the loss of E2F affects cell proliferation during oncogenic stimuli has not been studied. Given that most models emphasize the prominent role of E2F in proliferation of tumor cells, this is an important question to be addressed.

*Drosophila* has proven to be an excellent model to investigate cancer-causing genes. This is illustrated, for example, by studies of the recently identified the Hippo tumor suppressor pathway. The cellular functions of the Hippo pathway are to restrict cell proliferation and promote apoptosis (for review see: [19]–[22]). The core components of the pathway are the protein kinases Warts (Wts), Hippo (Hpo) and Mob as tumor suppressor (Mats). Salvador (Sav) serves a scaffold for Wts and Hpo. Assembly of an active complex of the four negative regulators Wts/Hpo/Mats/Sav is accompanied by mutual phosphorylation and leads to activation of the Wts kinase. Once active, Wts phosphorylates and inactivates the transcriptional co-activator Yorkie (Yki) by excluding Yki from nucleus. Yki is thus far the most downstream component of the pathway. In the absence of Wts-dependent phosphorylation, Yki enters the nucleus where it requires transcription factors to be recruited to the promoter of the Hippo pathway target genes. Thus far, only the lone TEAD/TEF protein family member in *Drosophila*, Scalloped, has been shown to interact with Yki [23],[24] while the Yki mammalian homolog YAP binds to a variety of transcriptional factors and modulates their activity. Among Hippo pathway targets are genes that promote cell proliferation and genes that inhibit apoptosis such as *cyclin E*, microRNA *bantam,* and *diap1*. Inactivation of any negative regulator of Hippo pathway signaling, or overexpression of the positive regulator Yki, stimulates additional cell divisions by increasing the proliferation rate of actively dividing cells, delaying the cell cycle exit, and simultaneously protecting cells from apoptosis. Failure to exit the cell cycle on time gives rise to inappropriate proliferation of Hippo pathway mutant cells. Since patterns of cell proliferation are normal in *dDP* mutants and in *de2f1 de2f2* double mutants, these combinations provide us with an opportunity to determine when and where proliferation driven by the potent oncogene Yki is dependent on dE2F family members. In this work, we show that the loss of E2F function produces a distinctly different result in actively dividing cells and in cells that proliferate inappropriately due to the failure to exit the cell cycle. Inactivation of the entire dE2F family in actively dividing cells has only a subtle effect on the ability of Yki to increase the rate of cell division. In contrast, the loss of E2F function fully blocks inappropriate proliferation of these cells. Thus, our work uncovers the *in vivo* requirement for E2F function during oncogenic proliferation driven by Yki, specifically at the point when cells normally exit the cell cycle and enter quiescence.

## Results

### Yki-Driven Proliferation of Actively Dividing Cells in the Wing Imaginal Disc Does Not Require E2F Mediated Control

**Figure 1:**
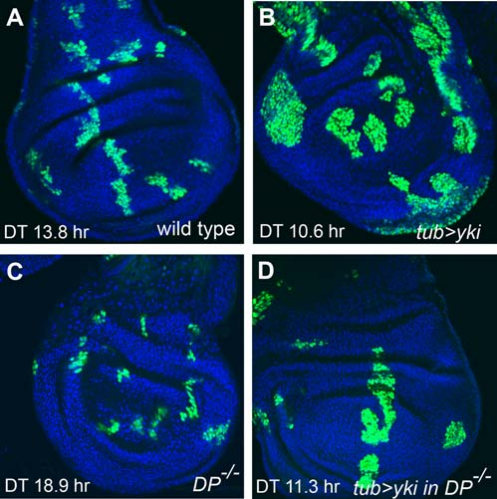
Inactivation of the dE2F family does not block Yki-induced proliferation in actively dividing cells. Clones of cells of four different genotypes marked with GFP (green) were induced simultaneously with the MARCM technique and allowed to grow for the same period of time. Cells were visualized by staining with DAPI (blue). Population doubling time (DT) is shown for each genotype. Data were collected from 28 clones for wild type, 28 clones for *tub*>*yki*, 66 clones for *DP^−/−^*, and 33 clones for *tub*>*yki* in *DP^−/−^*. Average clone areas are 9,206±613 pixels for wild type; 18,491±1,780 pixels for *tub*>*yki*; 2,814±279 pixels for *DP^−/−^* and 12,909±1,263 pixels for *tub*>*yki* in *DP^−/−^*. (A) Control clones induced with a wild type *FRT42D* chromosome. (B) Clones of wild type cells that overexpress *yki* contain more cells than control in (A). (C) Clones of *dDP* mutant cells. (D) Clones of *dDP* mutant cells that overexpress *yki* contain more cells than clones of *dDP* mutant cells (C) or control clones (A).

We initially used a *dDP* mutation to determine whether Yki requires dE2F mediated control to drive cell proliferation. dDP is an obligatory heterodimeric partner of both dE2F1 and dE2F2, and the loss of *dDP* has been shown to functionally inactivate both *de2f1* and *de2f2* [15]. Importantly, *dDP* single mutant or *de2f1 de2f2* double mutant animals survive until late pupal stages and exhibit normal patterns of cell proliferation [17],[18]. Thus, the use of these mutant combinations allowed us to minimize indirect cell cycle effects produced when dE2F1 function alone is inactivated. The MARCM technique [25] was employed to generate clones of wild-type cells, wild-type cells overexpressing *yki*, *dDP* mutant cells, and *dDP* mutant cells that overexpress *yki* in the larval wing imaginal disc. All clones were marked with GFP, induced simultaneously, and allowed to grow for the same period of time (Figure 1). At this stage of development, the majority of cells in the wing disc are asynchronously dividing and therefore the rates of cell proliferation can be accurately measured. As expected, overexpression of *yki* accelerated the cell cycle progression of wild type cells (Figure 1A–B). The median population doubling time in *yki* overexpressing clones was 10.6 hr. This was faster than that of the wild type population, which was 13.8 hr. We found that a *dDP* mutation did not significantly affect the ability of *yki* to increase rates of cell division (Figure 1C–D). The median population doubling time in clones of *dDP* mutant cells overexpressing *yki* was 11.3 hr, indicating that these cells were still proliferating faster than wild type cells. Thus, this result suggests that E2F mediated control is not required for Yki induced proliferation in actively dividing cells.

### The Loss of E2F–Dependent Control Blocks Yki-Driven Inappropriate Proliferation in the Cells Posterior to the SMW in the Eye Imaginal Disc

**Figure 2:**
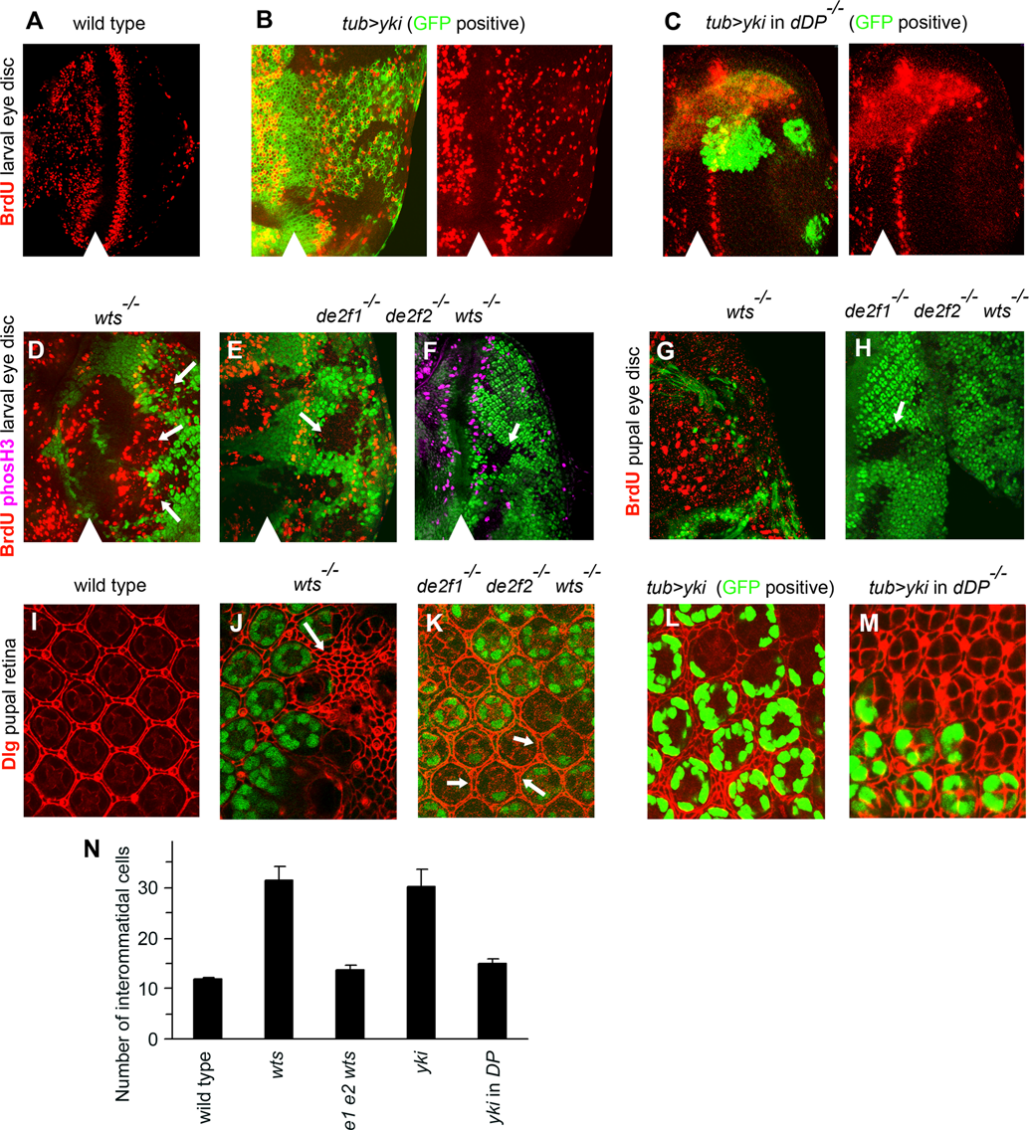
Yki-driven proliferation of cells posterior to the SMW is blocked in the absence of the dE2F family. (A) The pattern of S phases in the wild type eye discs as revealed by BrdU labeling. Position of the morphogenetic furrow (MF) is shown by arrowhead. Posterior is to the right. Wild type cells asynchronously proliferate anterior to the MF, arrest in G1 in the MF and undergo a synchronous S phase in the second mitotic wave (SMW) posterior to the MF. (B–C) Clones of wild type (B) and *dDP* mutant (C) cells overexpressing *yki* were generated with the MARCM system and marked with GFP (green). Clones in (B) were generated with the *ey*-FLP while clones in (C) were generated with the *hs*-FLP. (B) Posterior to the SMW, wild type cells that overexpress *yki* fail to exit the cell cycle and proliferate inappropriately as evident by the appearance of BrdU positive cells. (C) In contrast, *yki* is unable to induce inappropriate proliferation of *dDP* mutant cells posterior to the SMW. Note, that *dDP* mutant cells that overexpress *yki* show a normal pattern of BrdU incorporation in the SMW but do not incorporate BrdU posterior to the SMW. (D–M) Clones of mutant cells of different genotypes were generated with *ey*-FLP and the mutant tissue was distinguished by the lack of GFP (green). (D–F) Mosaic larval eye discs were labeled with BrdU (red) to detect the S phases (D and E) or stained with anti-phosH3 (magenta) to visualize mitoses (F). (D) *wts* mutant cells fail to exit the cell cycle posterior to the SMW and undergo inappropriate proliferation, which is evident by the persistence of BrdU incorporation (pointed by arrows). (E–F) In contrast, inappropriate proliferation posterior to the SMW is strongly reduced in *de2f1 de2f2 wts* triple mutant cells as judged by the absence of cells in S phase (red in E) or in mitosis (magenta in F) (pointed by arrows). (G–H) BrdU incorporation (red) in 12 hr pupal eye discs. (G) *wts* mutant cells continue unscheduled proliferation during early pupal development while wild type cells remain fully quiescent as revealed by BrdU labeling. (H) Inappropriate BrdU incorporation is absent in clones of *de2f2 de2f1 wts* triple mutant cells (a clone is pointed by arrow). (I–M) Pupal retina at 48 hr APF stained with anti-Discs large protein (Dlg) (red) to visualize cell outlines. (I) Wild type retina contains a single layer of interommatidial cells between ommatidial clusters. (J) Inappropriate proliferation of *wts* mutant cells posterior to the SMW and resistance of these cells to the developmental apoptosis during the pupal stage gives rise to the dramatic excess of interommatidial cells (pointed by arrow). (K) In contrast, the number of interommatidial cells is significantly reduced in *de2f1 de2f2 wts* triple mutant tissue (indicated by arrows). (L–M) The MARCM technique was used to overexpress *yki* in wild type (L) or in *dDP* mutant cells (M). A *dDP* mutation dramatically reduces supernumerary interommatidial cells which arise when *yki* is expressed. (N) Quantification of the number of interommatidial cells in pupal retina shown in (J–M). Data were collected from 23 ommatidia clusters for wild type, 11 ommatidia clusters for *wts*, 24 ommatidia clusters for *de2f1 de2f2 wts*, 12 ommatidia clusters for *tub*>*yki* and 17 ommatidia clusters for *tub*>*yki* in *DP^−/−^*. The following abbreviations were used: *e1 e2 wts* corresponds to *de2f1 de2f2 wts*; *yki* corresponds to *tub*>*yki* and *yki* in *DP* corresponds to *tub*>*yki* in *DP^−/−^*. Error bars represent standard deviations. Note that in comparison to the wild type tissue there is a small excess of interommatidial cells in *de2f1 de2f2 wts* triple mutant tissue and in *dDP* mutant tissue that overexpresses *yki*. This is likely due to the failure to execute normal pupal developmental apoptosis in these cells.

In the eye imaginal disc, Hippo pathway mutant cells delay the cell cycle exit and undergo inappropriate proliferation. To determine the effect of the loss of E2F mediated regulation in these settings, we examined the effect of *yki* overexpression in clones of wild type or *dDP* mutant cells during cell cycle exit. In a wild type eye disc, BrdU labeling reveals a narrow stripe of S phase cells posterior to the morphogenetic furrow (MF), referred to as the Second Mitotic Wave (SMW) (Figure 2A). Cells within the MF are arrested in G1 and therefore do not incorporate BrdU. Posterior to the SMW, cells exit the cell cycle and differentiate. In contrast, *yki* overexpressing cells (GFP positive) failed to undergo cell cycle exit and continued proliferating [26], as shown by the appearance of BrdU positive cells posterior to the SMW (Figure 2B). Strikingly, no ectopic BrdU incorporation was observed when *yki* was overexpressed in clones of *dDP* mutant cells, which are marked by the presence of GFP (Figure 2C). This indicates that although E2F function is unnecessary for Yki to increase rates of cell proliferation in actively dividing cells, Yki is dependent on the presence of dE2F/dDP activity to drive cells into inappropriate cell cycles posterior to the SMW.

To further confirm this conclusion, we examined cell proliferation when both *de2f1* and *de2f2* were genetically ablated while the Hippo pathway was inactivated by a *wts* mutation. Clones of *de2f2* single, *de2f1 wts* double, and *de2f2 de2f1 wts* triple mutant cells were simultaneously generated in the same eye imaginal disc using the *ey-*FLP/FRT technique. The triple mutant tissue could be distinguished from the neighboring wild type tissue by the complete lack of GFP. Similar to *yki* overexpressing cells, *wts* mutant cells failed to exit the cell cycle and proliferated inappropriately posterior to the SMW [27],[28] (Figure 2D). Additionally, *wts* mutant cells continue cell divisions during early pupal development when wild type cells are quiescent (Figure 2G). The inappropriate proliferation during larval and pupal stages gives rise to a surplus of interommatidial cells. During pupal eye development, the excess of interommatidial cells is eliminated by a wave of apoptosis. However, since *wts* mutants are defective in normal apoptosis in the eye, these supernumerary cells remain and can be visualized in 48 hr old pupal retina as extra layers of cells between adjacent ommatidial clusters (Figure 2J). Consistent with the results of the overexpression of *yki* in *dDP* mutant cells (Figure 2C), clones of *de2f2 de2f1 wts* mutant cells posterior to the SMW were largely devoid of BrdU incorporation and mitoses, the latter were detected by the appearance of phosphorylated histone H3 (phosH3), (Figure 2E–F). No S phases were detected in the triple mutant combination at 12 hr after puparium formation either (Figure 2H), a time point when *wts* mutant cells continue inappropriate proliferation (Figure 2G). Furthermore, examination of pupal retinas revealed that clones of *de2f1 de2f2 wts* triple mutant cells (Figure 2K) or *dDP* mutant cells that overexpress *yki* (Figure 2M) no longer contain an abnormally large number of supernumerary interommatidial cells which are otherwise found in clones of *wts* mutant cells (Figure 2J) or in clones of cells that overexpress *yki* (Figure 2L). To measure the extent to which the loss of dE2F function reduced the number of interommatidial cells in *wts* mutant tissue, we counted the number of secondary, tertiary, and bristles cells per each ommatidial hexagon. Clones of *wts* mutant tissue in pupal retinas contained an average of 31.6±2.6 cells, which was significantly higher than 12.0±0.1 cells found in wild type tissue (Figure 2N). However, the regions that were triple mutant for *de2f1 de2f2 wts* had only 13.7±1.0 cells. Similarly, a *dDP* mutation significantly reduced the number of cells in clones that overexpress *yki* from 30.3±3.3 down to 15.0±1.0 (Figure 2N). These reductions are consistent with the observations that these cells fail to proliferate posterior to the SMW. We further emphasize that the inability of *wts* mutant cells, or *yki* overexpressing cells, to undergo inappropriate proliferation in the absence of E2F control is not merely a consequence of non-specific cell cycle defects due to inactivation of the dE2F family. Most cell proliferation occurs normally in *de2f1 de2f2* double mutants [18] or *dDP* mutants [17] and, as shown here, a *dDP* mutation did not prevent *yki* from increasing rates of cell division in asynchronously dividing cells of the wing disc (Figure 1).

**Figure 3:**
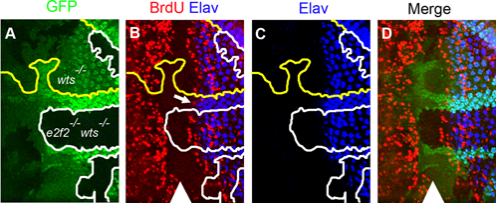
Loss of *de2f2* does not affect the *wts* mutant phenotype in the eye. Clones of mutant cells were generated with *ey*-FLP. Position of the morphogenetic furrow (MF) is marked by a white arrowhead in B and D. Posterior is to the right. (A) *de2f2* and *wts* are on two separate chromosomal arms. Therefore following expression of *ey-*FLP, clones of cells of four different genotypes are generated (wild type, *de2f2* mutant, *wts* mutant and *de2f2 wts* double mutant). *de2f2 wts* double mutant tissue is marked by the lack of GFP (green), is labeled *e2f2^−/−^ wts^−/−^* in (A) and is outlined by a white line in (A–C). *wts* single mutant tissue is distinguished by a reduced intensity of GFP (green) and an increased spacing between ommatidial clusters (marked by ELAV). An example of *wts* mutant tissue is labeled *wts^−/−^* in (A) and is denoted by yellow outline in (A–C). Wild type tissue is distinguished by the strongest level of GFP (green). An example of the wild type tissue is found between the yellow and white line. (B–D) Mosaic larval eye discs were labeled with BrdU (red) to detect cells in S phase (B,D) and ELAV (blue) to identify position of ommatidial clusters (B–D). Posterior to the MF, wild type cells undergo a single round of S phases in the second mitotic wave (SMW) (denoted by white arrow in B). In contrast, inappropriate BrdU (red) incorporation posterior to the SMW was detected in both *wts* and *de2f2 wts* mutant cells (B,D). (C) As a result of this inappropriate proliferation, spacing between ommatidial clusters is increased in both *wts* and *de2f2 wts* mutant tissue in comparison to wild type tissue. A merged image is shown in D.

Next, we wished to determine whether both dE2F family members are equally important in *wts* mutant cells to undergo inappropriate proliferation. To address this question we compared the S phases posterior to the SMW in clones of *de2f2 wts* double mutant cells with that of *wts* single mutant cells in the same eye imaginal disc. Previous analysis revealed that patterns of cell proliferation are normal in clones of *de2f2* mutant cells [29]. The *de2f2 wts* double mutant cells were marked by the lack of GFP, *wts* single mutant cells were distinguished by an intermediate GFP signal and by the increased spacing between ommatidial clusters, while the wild type cells had the highest intensity of GFP (Figure 3A). The loss of *de2f2* had no effect on inappropriate S phases posterior to the SMW in *wts* mutant cells, as the phenotype of *de2f2 wts* mutant cells was indistinguishable from the phenotype of *wts* mutant cells (Figure 3B–D). Additionally, the spacing between adjacent ommatidial clusters (marked with Elav) is increased in clones of *de2f2 wts* double mutant cells when compared to that of wild type cells. Such increase reflects the appearance of additional interommatidial cells in the *wts* mutant tissue [27]. Taken together with the results described above, this indicates that *de2f2* is not important for the phenotype of *wts* mutant cells and that *de2f1* is required for inappropriate proliferation of *wts* mutant cells. Consistently, no ectopic S phases were detected in *de2f1 wts* double mutant clones (data not shown).

**Figure 4:**
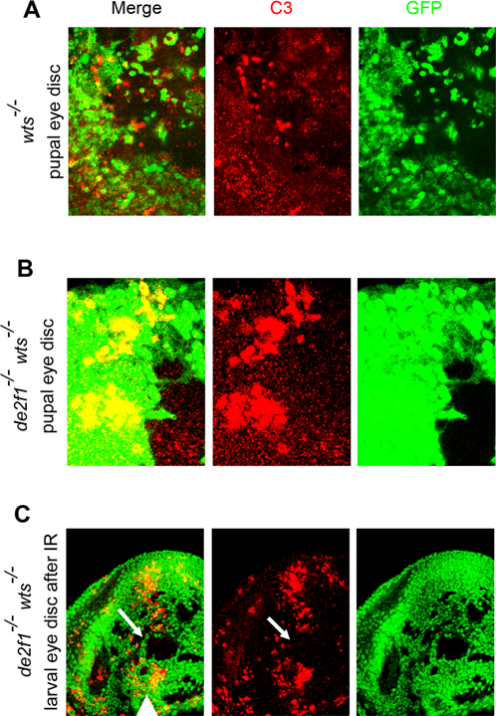
Loss of *de2f1* does not affect resistance to DNA damage and pupal developmental apoptosis in *wts* mutants. In all panels, clones were generated with the *ey*-FLP/FRT technique and mutant tissue is distinguished by the absence of GFP (green). (A–B) The pupal eye discs at 30 hr APF containing clones of *wts* mutant (A) and *de2f1 wts* double mutant (B) cells were stained with anti-Cleaved Caspase3 (C3) antibody (red) to detect apoptotic cells. In the pupal eye discs, developmentally regulated apoptosis is abundant in wild type cells (green) but is largely absent in *wts* mutant tissue (lack of green) and in *de2f1 wts* double mutant tissue indicating that *de2f1 wts* double mutant cells, like *wts* mutant cells, are protected from the cell death. (C) DNA damage induced apoptosis following irradiation was detected with anti-C3 antibody (red). There is an extensive apoptosis in wild type tissue. In contrast, *de2f1 wts* double mutant cells are protected from apoptosis after DNA damage. An example of *de2f1 wts* double mutant tissue is pointed by arrow. The morphogenetic furrow is marked by the arrowhead.

In addition to delaying the cell cycle exit, the loss of Hippo pathway protects cells from both developmental and stress induced apoptosis [19]–[22]. Although a mutation in *de2f1* blocked inappropriate proliferation in Hippo pathway mutant cells, *de2f1 wts* double mutant cells, like *wts* single mutant cells, were fully resistant to a wave of apoptosis that normally occurs during pupal eye development (Figure 4A–B) or to DNA damage induced apoptosis in the larval eye disc following irradiation (Figure 4C). Thus, resistance to apoptosis, a hallmark of inactivation of Hippo pathway, remains unaffected by the loss of *de2f1*. This sustained resistance to apoptosis is a likely explanation for the slight increase of the number of interommatidial cells in *de2f1 de2f2 wts* mutant tissue and in *dDP* mutant cells overexpressing *yki* in comparison to wild type (Figure 2N). From these data we concluded that *de2f1* is specifically required in Hippo pathway mutant cells to delay the cell cycle exit and sustain inappropriate proliferation posterior to the SMW.

### The Loss of E2F–Mediated Control Does Not Block Induction of Yki Target Genes

**Figure 5:**
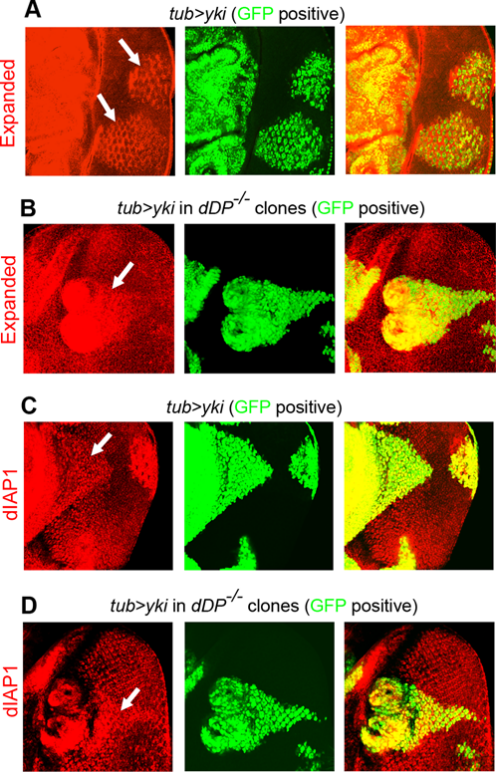
*dDP* mutation does not block *yki-*dependent induction of its target genes, dIAP1 and Expanded. The MARCM system was used to drive *yki* overexpression in wild type (A, C) or in *dDP* mutant cells (B, D) of the larval eye disc. Cells that express *yki* are marked with GFP (green). Merge images are on the right. *yki* overexpression induces Expanded (A) and dIAP1 (C) expression (pointed by arrows) in wild type cells. Inactivation of the dE2F family in *dDP* mutant cells does not significantly affect *yki*-dependent induction of Expanded (B) and dIAP1 (D) (pointed by arrows). Images in (B and D) show the same clone that was double stained with dIAP1 and Expanded, while images in (A and C) represent two different clones stained singularly with Expanded (A) and dIAP1 (C).

A trivial explanation for the lack of inappropriate proliferation in *de2f2 de2f1 wts* is that inactivation of dE2F family members renders Yki inactive in these cells. To directly address this question we examined whether Yki is capable of inducing its target genes in *dDP* mutant cells. *Drosophila* inhibitor of apoptosis, dIAP1, has been recently shown to be a direct Yki transcriptional target [23],[24] and, together with Expanded [30], are commonly used to accurately assess the activity of the Hippo pathway. Notably, upregulation of Expanded and dIAP1 following *yki* overexpression was observed in wild type cells (Figure 5A, 5C) and to almost the same extent in clones of *dDP* mutant cells (Figure 5B, 5D). This suggests that the loss of E2F regulation does not prevent induction of at least two well established Yki targets.

**Figure 6:**
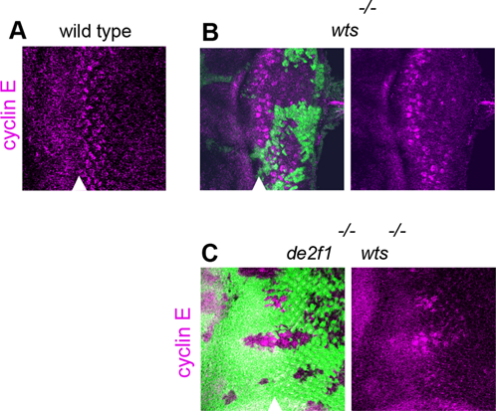
Loss of *de2f1* does not block induction of cyclin E in *wts* mutant cells. Clones of mutant cells were generated with *ey*-FLP and distinguished by the lack of GFP (green). (A) In wild type eye imaginal discs, cyclin E (magenta) expression is elevated within and immediately posterior to the morphogenetic furrow (MF). In *wts* mutant cells (B) and in *de2f1 wts* double mutant cells (C) cyclin E is expressed further posterior. Position of MF is shown by arrowhead. Posterior is to the right.

Next, we investigated the expression of cyclin E since it is an E2F target [2] and is also considered to be a critical target of the Hippo pathway [19]–[22]. Clones of *wts* single and *de2f1 wts* double mutant cells were generated. As shown in Figure 6, the level of cyclin E was elevated in *wts* mutant cells and in *de2f1 wts* double mutant cells. Thus, the observation that induction of multiple Yki target genes is not compromised by the loss of E2F control implies that Yki remains active in dE2F deficient cells. Such a conclusion is in agreement with the resistance to apoptosis of *de2f1 wts* mutant cells, further evidence that Yki is fully functional in the absence of dE2F family.

### Inactivation of the Hippo Pathway Leads to Elevation of E2F Activity

**Figure 7:**
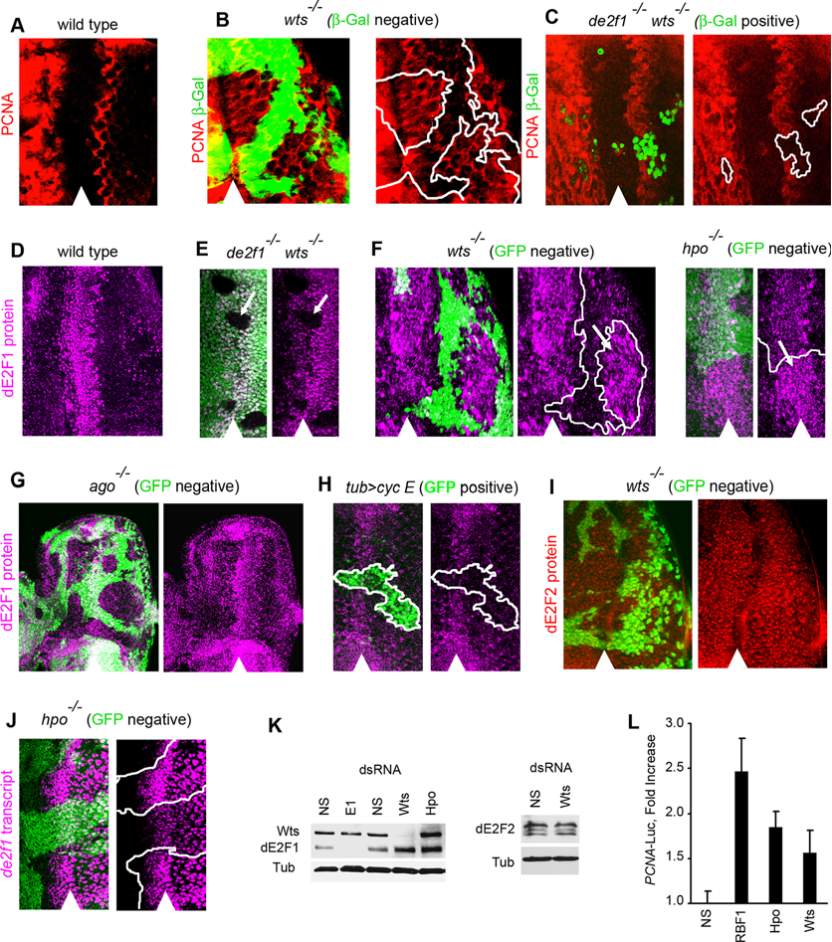
Elevated E2F activity in cells with inactivated Hippo pathway. Clones of mutant cells were induced with *ey*-FLP. Position of the morphogenetic furrow (MF) is shown by arrowhead. Posterior is to the right. (A–C) Expression of the E2F reporter, *PCNA*-GFP, (red) in the wild type eye disc (A), or in the eye discs containing clones of *wts* mutant tissue (B) and *de2f1 wts* double mutant tissue (C). (A) In wild type disc, the E2F reporter is expressed in a narrow stripe (red) immediately posterior to the MF, preceding S phase entry into the second mitotic wave (SMW). (B) In *wts* mutant cells which are marked by the absence of β-Gal (green), the E2F reporter is inappropriately expressed in the posterior region of the eye disc. Mutant tissue is outlined. (C) Inappropriate expression of the E2F reporter in the posterior region of the eye disc is absent in *de2f1 wts* double mutant cells. Note, that clones of *wts de2f1* double mutant cells were marked with β-Gal (green) produced from the *de2f1^729^* mutant allele. *de2f1 wts* double mutant tissue is outlined. (D) Endogenous dE2F1 (magenta) is expressed within the MF in a wild type disc as revealed by anti-dE2F1 antibody. (E–G, I) Clones of mutant cells were induced with *ey*-FLP and mutant tissue is identified by the lack of GFP (green). (E) The anti-dE2F1 antibody is highly specific as the staining is absent in *de2f1* mutant tissue (lack of green in E and pointed by the arrow). (F) *wts* and *hpo* mutant cells have an increased level of dE2F1 within the MF and inappropriately express dE2F1 posterior to the MF. Examples are pointed by the arrows. Position of mutant tissue is outlined. (G) Expression of endogenous dE2F1 protein (magenta) is unaffected in *ago* mutant cells in larval imaginal eye discs. (H) cyclin E was expressed ectopically in wild type mitotic clones using the MARCM system. Ectopic expression of cyclin E fails to elevate level of dE2F1 protein (magenta) posterior to the MF. Cells that express cyclin E are marked with GFP (green) and are outlined. (I) Endogenous dE2F2 protein (red) is expressed ubiquitously throughout the eye disc. Level of dE2F2 protein remains the same in both *wts* mutant and wild type tissue. (J) *de2f1* is transcriptionally induced in *hpo* mutant cells as revealed by the *de2f1* enhancer trap allele, *de2f1^729^*. *de2f1^729^* contains the *lacZ* insertion into the endogenous *de2f1* gene. The *lacZ* expression reflects transcription from the *de2f1* promoter [34],[35]. Staining with anti-β-Gal antibody (magenta) was used to reveal expression of the *lacZ* gene in *de2f1^729^*. (K) SL2 cells were treated with nonspecific (NS), dE2F1 (E1), Warts (Wts) and Hippo (Hpo) dsRNA to deplete the corresponding proteins by RNAi. Cell extracts were analyzed by Western blot using antibody specific for Wts, dE2F1 and dE2F2. Depletion of Wts and Hpo shows an increase in the level of dE2F1 protein. In contrast, the dE2F2 protein level is not affected. The same blots were re-probed with anti-Tubulin antibody to control for equal loading. (L) Endogenous E2F activity is elevated in Hpo or Wts depleted SL2 cells. SL2 cells were incubated with non-specific (NS), RBF1, Hpo, and Wts dsRNAs for 4 days to deplete the corresponding proteins. On day 4, the E2F reporter (*PCNA*-luc) was transfected into the depleted cells and the luciferase activity was measured 2 days later to determine the level of the endogenous E2F activity in these cells. The pIE-LacZ plasmid was co-transfected to normalize for transfection efficiency. Results depict the mean of three experiments. Unpaired Student's *t*-Test assuming equal variance concluded that the increase of *PCNA-*luc reporter activity in RBF1, Hpo and Wts depleted cells was statistically significant from the NS control. RBF1 and Hpo depleted cells had a p-Value <0.001. Wts depleted cells had a p-Value <0.03.

To further elucidate the role of *de2f1* in Hippo pathway mutant cells, we used a *PCNA*-GFP reporter [31] to accurately measure E2F activity in clones of *wts* mutant cells. In a wild type eye disc, the *PCNA*-GFP reporter is expressed in a narrow stripe of cells prior to S phase entry in the SMW and is absent in the posterior region of the eye (Figure 7A). In contrast, *wts* mutant cells failed to downregulate the expression of the *PCNA-*GFP reporter posterior to the SMW (Figure 7B). This indicates that these cells have an abnormally high E2F activity. Importantly, the high E2F activity is due to *de2f1* because the *PCNA*-GFP reporter is no longer expressed in *de2f1 wts* double mutant cells posterior to the SMW (Figure 7C). The finding that *wts* mutant cells have a high E2F activity posterior to the SMW is unexpected since the activator dE2F1, which provides the pattern of expression of the *PCNA*-GFP reporter in the eye disc [31], is normally downregulated in these cells (Figure 7D and [15]). Therefore, we examined the expression of dE2F1 in clones of *wts* and *hpo* mutant cells using a highly specific dE2F1 antibody (Figure 7E). In contrast to the wild type dE2F1 pattern, the expression of dE2F1 was highly abnormal in *wts* and *hpo* mutant cells (Figure 7F). First, the level of dE2F1 was elevated in cells that normally express dE2F1 within the MF. Second, dE2F1 was ectopically expressed in cells posterior to the MF. Thus, Hippo pathway mutant cells that inappropriately proliferate posterior to the SMW have an elevated E2F activity which is likely due to a high level of dE2F1.

To determine whether the high level of dE2F1 is a specific response to inactivation of the Hippo pathway or an indirect consequence of inappropriate cell proliferation posterior to the SMW, we examined the pattern of dE2F1 expression in clones of *archipelago* (*ago*) mutant cells. *ago* mutant cells, like *wts* or *hpo* mutant cells, fail to exit the cell cycle and proliferate posterior to the SMW [32]. In contrast to the abnormally high expression of dE2F1 in Hippo pathway mutant cells, the level of dE2F1 was not elevated in clones of *ago* mutant cells posterior to the SMW (Figure 7G). Furthermore, expression of cyclin E, which is sufficient to drive quiescent cells posterior to the SMW into the cell cycle [33],[34], did not result in an increase of the level of dE2F1 (Figure 7H). Finally, the level of another dE2F family member, dE2F2, is unaffected in *wts* mutant cells (Figure 7I). Thus, we concluded that dE2F1 is specifically upregulated following inactivation of the Hippo pathway.

As a further characterization, we tested whether *de2f1* is transcriptionally induced in Hippo pathway mutant cells. The *in vivo* activity of the *de2f1* promoter in *hpo* mutant cells was examined using an enhancer trap allele, *de2f1^729^*. The *de2f1^729^* allele has been extensively used as an accurate measurement of *de2f1* transcription [34]–[36]. This allele carries a *lacZ* gene inserted within the endogenous *de2f1* gene. Therefore, the production of β-galactosidase reflects transcriptional activity at the *de2f1* promoter. Clones of *hpo* mutant cells were induced in the eye disc and the expression of the *lacZ* gene from the *de2f1^729^* allele was compared between the *hpo* mutant and adjacent wild type cells. Through detection of immunofluorescence, we found a higher level of β-Gal in *hpo* mutant cells than in wild type cells within the MF; thus, indicating that *de2f1* transcription was induced (Figure 7J).

Finally, we depleted SL2 tissue culture cells of Hpo and Wts by RNA interference, (RNAi), and examined what effect this had on the level of dE2Fs by western blot analysis. Consistent with the effects seen in eye imaginal discs, the dE2F1 protein level was markedly increased in cells deficient of either Wts or Hpo, while the dE2F2 protein level remained constant (Figure 7K). To further biochemically characterize the properties of Hpo and Wts depleted cells we transiently transfected these depleted cells with an E2F reporter, *PCNA*-luc. Depletion of RBF1, the *Drosophila* pRB homolog, elevated the expression of the reporter by 2.5 fold in comparison to control treated cells (Figure 7L). Noticeably, the reporter was induced approximately 2 fold in Hpo and in Wts depleted cells, which is similar to the level seen in cells deficient of RBF1, the endogenous inhibitor of dE2F1. We concluded that inactivation of the Hippo pathway increases the level of dE2F1 and, more importantly, elevates E2F activity both *in vivo* and *in vitro*.

## Discussion

Current models emphasize the importance of the E2F transcription factor in cell cycle control as one of the key downstream targets of the pRB tumor suppressor protein. Although E2F activity is rate limiting for S phase entry in tissue culture cells, ablation of the entire pool of *Drosophila* E2F is permissive for cell proliferation *in vivo* and only marginally affects animal development. However, it is unknown whether oncogene driven cell proliferation would also be insensitive to the loss of the entire dE2F family. This is an important conceptual point because unrestrained proliferation is a central property of a cancer cell, and this unrestrained proliferation is thought to be the result of deregulated E2F activity [5].

In this report, we addressed the role of the dE2F family members in cell proliferation following inactivation of the recently identified Hippo tumor suppressor pathway. In order to minimize any possible non-specific cell cycle effects seen in the presence of a *de2f1* mutation, we have taken advantage of the previous observation that the complete ablation of E2F function in either *de2f1 de2f2* double mutants or *dDP* single mutants is permissive for cell proliferation [15],[17],[18]. Our results strongly argue that the effect of the loss of E2F function on proliferation of Hippo pathway mutant cells is distinctly different in actively dividing cells and in cells undergoing unscheduled proliferation posterior to the SMW. In actively dividing cells that overexpress the pro-oncogene *yki*, a positive effector of the Hippo pathway, inactivation of the dE2F family has a minimal effect on cell proliferation; as Yki is capable of dramatically accelerating the rate of cell cycle progression of *dDP* mutant cells. Similarly, clones of cells which lack *de2f1*, *de2f2*, and *wts* (a negative regulator of Yki) appear to be relatively large; however, the quantification of a population doubling time in these mutant cells is technically inaccurate due to the requirement of two independent recombination events to generate triple mutant clones. Elimination of E2F function does not abolish Yki-dependent transcription, thus, we suggest that an elevated level of Yki target genes such as *cyclin E* and others may account for the accelerated proliferation of *dDP* mutant cells. Indeed, previous studies have shown that transient expression of *cyclin E* is sufficient to increase the rate of DNA synthesis of *dDP* mutant cells in the eye disc [15]. Thus, another conclusion that we drew from these results is that proliferation defects of *dDP* mutant cells are essentially rescued by Yki overexpression. This idea is consistent with the notion that Yki and the dE2Fs appear to share some common targets such as *cyclin E*. A caveat to this explanation is that for the exception of *diap1*, it is not known what putative targets are directly regulated by Yki. Secondly, since Yki fails to induce an E2F-reporter in the absence of *de2f1*, this suggests that Yki does not generally rescue E2F-dependent transcription in *dDP* mutant cells, but rather increases expression of a limited set of shared targets. Discerning how Yki overexpression accelerates the rate of proliferation in the absence of dE2F and how the interplay between Yki and dE2F occurs at common targets will be important directions in future studies.

In striking contrast to the results of inactivation of the entire dE2F family in actively dividing cells, we find that E2F function is required during Yki-driven unscheduled proliferation in otherwise quiescent cells posterior to the SMW in the eye imaginal disc. Overexpression of *yki* or inactivation of negative regulators of the Hippo tumor suppressor pathway, such as *wts*, renders cells of the eye imaginal disc refractory to the cell cycle exit signals and, as a result, cells continue to proliferate inappropriately [19],[21]. These abnormal cell cycles are fully blocked when E2F function is eliminated either by a mutation in the *dDP* gene or by combined ablation of both *de2f1* and *de2f2* genes. This conclusion is supported by the complete absence of cells in S phase or in mitosis posterior to the SMW in mutant clones. Furthermore, loss of E2F function in clones of *wts* mutant cells or in clones of cells that overexpress *yki* significantly reduces the number of supernumerary interommatidial cells that primarily arise due to inappropriate proliferation during larval and early pupal development. Interestingly, this reduction is very similar to that seen in clones of *expanded* mutant cells, an upstream negative regulator of Yki, albeit the molecular mechanism is distinctly different. Unlike *de2f1 de2f2 wts* triple mutants, *expanded* mutant cells proliferate inappropriately posterior to the SMW [37]. However, the supernumerary interommatidial cells are largely removed during the wave of developmental pupal apoptosis, while *de2f1 de2f2 wts* triple mutant cells are fully protected from cell death.

The results described here highlight the specific requirement for dE2F to maintain a proliferation potential in cells with high Yki activity posterior to the SMW. We emphasize that the loss of E2F control is permissive for cell proliferation in actively dividing wild type cells, as well as in actively dividing cells that overexpress *yki*. However, inactivation of the dE2F family fully prevents inappropriate divisions of Hippo pathway mutant cells that have failed to exit the cell cycle. While one could predict this result in the absence of *de2f1* alone, since cell proliferation is severely reduced in *de2f1* mutants, it was perhaps surprising to find that Yki-driven inappropriate proliferation posterior to the SMW is completely blocked by the total inactivation of the dE2F family. In this respect, these results are distinct from the predicted outcome of inactivation of other cell cycle regulators such as *cyclin E* or *cyclin A* on cell proliferation in Hippo pathway mutants. Mutations in these genes are likely to fully abrogate Yki induced proliferation in most, if not all, settings due to their fundamental roles in cell cycle regulation. In support of this distinction we note that the loss of the microRNA *bantam* has been shown to block Yki-driven proliferation in actively dividing cells of the wing disc, as well as cell proliferation during normal development [38],[39].

Why is Yki unable to drive cells into the cell cycle in the absence of E2F activity? One formal possibility is that Yki function is compromised in dE2F deficient cells posterior to the SMW. However, this seems unlikely since *de2f1 wts* double mutant cells, like *wts* single mutant cells, are fully protected from DNA damage induced apoptosis at larval stage and from developmental apoptosis in the pupal eye. Thus, inhibition of apoptosis, one of the key aspects of Yki function, remains unaltered. Consistently, Yki does induce its target genes, *diap1* and *Expanded*, in *dDP* mutant cells. Hence, Yki activity does not appear to be generally affected by the loss of E2F function. We also note that Yki-dependent induction of cyclin E (this work) and cyclin B (our unpublished observations) still occurs in *de2f1* deficient cells posterior to the SMW, yet these cells fail to proliferate posterior to the SMW. Thus, it remains a likely possibility that high cyclin E activity is capable of driving proliferation in actively dividing cells in the absence of dE2F, but not in cells during the cell cycle exit where cyclin E appears to require an assist from dE2F1 to sustain unscheduled cell proliferation. Although we cannot formally exclude the possibility that expression of some Yki target genes is deregulated in dE2F deficient cells, these results suggest that in the absence of dE2F, the Yki transcriptional program alone is insufficient to drive cell proliferation in otherwise quiescent cells posterior to the SMW. We emphasize that overexpression of dE2F1 is not sufficient to sustain proliferation in cells posterior to the SMW (for example see: [35],[40]) and that the phenotype of Hippo pathway mutant cells is likely to be a result of a cumulative effect of deregulation of a panel of Yki target genes. This is consistent with several studies that have shown that upon the cell cycle exit, cells become highly resistant to proliferative signals. For example, co-expression of dE2F1 and cyclin E is needed to bypass the cell cycle exit [40]. Similarly, combined ablation of two negative regulators of the cell cycle, RBF1 and the cdk2 inhibitor Dacapo, is required to prevent the exit from the cell cycle in the larval eye [41]. Thus, our results highlight the need for dE2F during inappropriate proliferation at the specific point when cells attempt to exit the cell cycle.

The Hippo pathway controls epithelial tissue growth by regulating the expression of genes that can promote cell proliferation and genes that can inhibit apoptosis. In humans, loss of expression of Lats1/2 (Wts homolog) [42] and mutations in *WW45/Sav* [43] and *Mob* (homolog of *mats*) [44] have been found in several tumor cell lines, while YAP expression is frequently elevated in cancers [45],[46]. Accordingly, mouse embryos lacking *WW45/Sav* display hyperplasia due to defects in cell cycle exit and terminal differentiation of epithelial progenitor cells [47]; while *Lats1* ^−/−^ knockout animals develop soft-tissue sarcomas and ovarian stromal cell tumors [48]. Additionally, in a transgenic mouse model, YAP activation in the liver induces hyperplasia followed by tumor formation [46],[49]. Thus, the Hippo pathway represents a frequent mutational target and the outcome of its deregulation is tumorigenesis in both mice and humans. Although the status of the pRB pathway has not been determined in these tumors, it is generally thought that inactivation of the pRB pathway is an obligatory event in most, if not all, types of tumors [5]. In this respect, it is particularly intriguing that the ablation of Hippo function leads to an increase in dE2F1 level and elevation of E2F activity. Since the Hippo pathway is highly conserved between flies and mammals, it would be interesting to determine whether expression of mammalian *E2f*s is also induced following inactivation of the Hippo pathway. In support of this possibility we note that ectopic expression of YAP in transgenic mice increases the level of a well known E2F target gene, *PCNA* [49]. A comparison of *de2f2 wts* and *de2f1 de2f2 wts* mutant clones revealed that *de2f1* is more important during inappropriate proliferation than *de2f2*. Importantly, the dE2F1 increase is not a coincidental result of an accelerated rate of proliferation in Hippo pathway mutant cells, since the level of dE2F1 is normal in clones of *ago* mutant cells, which, like *wts* mutants, proliferate posterior to the SMW. Although dE2F1 induction appears to be a result of a transcriptional response in *hpo* mutant cells and following overexpression of Yki (this work and [36]), whether the *de2f1* promoter is directly regulated by Yki is currently not known. Further experiments will be necessary to decipher the exact mechanism by which Yki exerts its effect on *de2f1* expression.

Finally, the data described here have a potential implication in cancer research. Inactivation of total E2F activity using a dominant negative form of E2F has been shown to impair re-entry into the cell cycle from quiescence in immortalized murine fibroblasts [9]. We find that a complete genetic ablation of the dE2F family fully blocks proliferation of Hippo pathway mutant cells posterior to the SMW. However, Hippo pathway mutant cells do not undergo a transient quiescence state during inappropriate proliferation since the mutant cells continuously express proliferation markers, such as phosH3, cyclin B, cyclin A, incorporate BrdU, and have a high level of an E2F reporter posterior to the SMW (this work and [27],[28],[43]). Thus, dE2F is required to sustain inappropriate cell proliferation specifically at the point when cells normally exit the cell cycle and enter quiescence. One implication of this result is that, at least, in the case of the inactivation of the Hippo pathway, the use of pharmacological E2F inhibitors might be beneficial in tumors in which cell cycle exit cues are induced even though these tumors do not necessarily respond to these signals and pass through temporary states of quiescence.

## Materials and Methods

### Fly Stocks and Mosaic Analysis

For mutant analysis, the following strong loss of function or null alleles were used:

*de2f1^729^*, *de2f2^c03344^*, *wts^X1^*, *hpo^MGH4^*, *yki^B5^*, *ago^1^*, and *dDP^a4^*.

Clones of homozygous mutant cells were generated with the *ey-*FLP/FRT technique. For clones of *ago^1^* mutant cells the following genotype was used:

*ey-FLP*; *ago^1^ FRT80B/P[Ubi-GFP] FRT80B*

For clones of double mutant cells of *de2f2 wts* the following genotype was used:

*ey-FLP*; *de2f2^c03344^FRT40A/P[Ubi-GFP] FRT40A*; *FRT82B wts^X1^/FRT82B P[Ubi-GFP]*

For clones of double mutant cells of *wts de2f1* the following genotype was used:

*ey-FLP*; *FRT82B de2f1^729^ wts^X1^/ FRT82B P[Ubi-GFP]*

Clones of triple mutant cells of *de2f1 de2f2 wts* were generated in:

*ey-FLP*; *de2f2^c03344^FRT40A/P[Ubi-GFP] FRT40A*; *FRT82B de2f1^729^ wts^X1^/FRT82B P[Ubi-GFP]*

Activity at the *de2f1* promoter in *hpo* mutant tissue was determined in larvae of the following genotype:

*ey-FLP*; *FRT42D hpo^MGH4^*/*FRT42D P[Ubi-GFP]*; *FRT82B de2f1^729^/*+

To determine the expression of the E2F reporter, *PCNA-GFP*, in *wts* mutant and in *de2f1 wts* double mutant cells the following genotypes were used:

*ey-FLP*; *PCNA-GFP/+; FRT82B wts^X1^/FRT82B P[arm-LacZ]*

*ey-FLP*; *PCNA-GFP/+; FRT82B de2f1^729^ wts^X1^/FRT82B P[arm-LacZ]*

Analysis of *yki* overexpression was done with the MARCM system [25] in larvae of the following genotypes:

*y w hs-FLP70 tub-GAL4 UAS-GFP-6XMyc.NLS*; *FRT42D tub-GAL80/FRT42D dDP^a4^*; *UAS-Yki/+*

*y w hs-FLP70 tub-GAL4 UAS-GFP-6XMyc.NLS*; *FRT42D tub-GAL80/FRT42D*; *UAS-Yki/+*

*y w hs-FLP70 tub-GAL4 UAS-GFP-6XMyc.NLS*; *FRT42D tub-GAL80/FRT42D*

*eyFLP UAS-GFP*; *tub-GAL4 FRT82B P[UAS-yki]/FRT82B tub-GAL80*

Analysis of cyclin E overexpression was done with the MARCM system [25] in:

*y w hs-FLP70 tub-GAL4 UAS-GFP-6XMyc.NLS; FRT42D tub-GAL80/FRT42D; UAS-cyclinE/+*

To determine the cell population doubling time, clones of *dDP* mutant cells were induced 48 hrs AED and discs were dissected and fixed 66–70 hrs later. A standard error of the mean was calculated for each genotype. To determine number of interommatidial cells for the pupal retinae, bristle, secondary, and tertiary cells for one ommatidium were counted. One ommatidium was defined as a single cluster of 4 cone cells, 2 primary cells, 3 bristle cells, 3 tertiary cells, and 6 secondary cells. A standard deviation of the mean was taken to determine significance. To measure the area per clone the histogram function in Adobe Photoshop was used and a standard error of the mean was taken to determine significance.

### Immunohistochemistry

Antibodies used were as follows: mouse anti-cyclinE 1∶20 (from B. Edgar), guinea pig anti-Expanded 1∶500 (from R. Fehon), mouse anti-Discs Large 1∶400 (DSHB), mouse anti-BrdU 1∶50 (Beckton Dickinson ), rabbit anti-dE2F1 1∶400 (from C. Seum), mouse anti-β-galactosidase 1∶30 (DSHB), rabbit anti-GFP 1∶200 (Invitrogen), rabbit anti-C3 (Cleaved Caspase3) 1∶100 (Cell Signaling), rabbit anti-dE2F2 1∶100, rabbit anti-phosH3 1∶175 (Upstate), and Cy3, Cy5 (Jackson Immunolaboratories) and Alexa488 (Invitrogen) conjugated anti-mouse and anti-rabbit secondary antibodies. Larval and pupal tissues were fixed in 4% formaldehyde for 30 minutes on ice, washed in phosphate-buffered saline, and then incubated with antibodies overnight at 4°C in phosphate-buffered saline, 10% normal goat serum, and 0.3% Triton-X100 as previously described [29]. To detect dE2F1 protein, fixation was adjusted to 40 minutes on ice and then treated as described above. To detect cyclin E protein in larval eye imaginal disc, PLP fixation was used and then the same protocol described above was used. To detect S phases, dissected larval or pupal eye discs were labeled with BrdU for 2 hrs at room temperature and then the eye discs were fixed overnight in 1.5% formaldehyde at 4°C. Apoptosis was measured in pupal eye discs 30 hrs APF. To measure apoptosis following DNA damage, larvae were exposed to 40 Grays of irradiation and then imaginal discs were dissected 4 hrs later.

### S2 Cell Manipulations

RNAi and transient transfections were done as described previously[29]. For western blot analysis, S2 cells were lysed in NP40 buffer, frozen at −80°C for 1 hr, thawed, spun down, and then boiled in protein sample buffer. Samples were resolved using SDS-PAGE on 7% gels, transferred to Immobilon-P membrane (Millipore), and incubated with the following antibodies: mouse anti-E7(tubulin) 1∶9000 (DSHB), guinea pig anti-Wts 1∶10,000 (From K. Irvine), rabbit anti-dE2F2 1∶2,000, guinea pig anti-dE2F1 1∶7,000 (from T. Orr-Weaver).
